# A rare but troublesome complication of cesarean section: the uterocutaneous fistula. Report of two cases and review of literature

**DOI:** 10.1515/crpm-2021-0057

**Published:** 2022-08-19

**Authors:** Rossana Cicinelli, Ettore Cicinelli, Francesco Crupano, Marina Vinciguerra, Bruno Lamanna, Antonella Vimercati

**Affiliations:** Unit of Obstetrics and Gynecology, University of Bari, Piazza Giulio Cesare, Policlinico, Bari, Italy

**Keywords:** cesarean section, uterine diseases, uterocutaneous fistula

## Abstract

**Objectives:**

The occurrence of a uterocutaneous fistula (UCF) as an uncommon and severe complication of caesarean section (CS). The aim of this study was to describe two cases of UCFs after CS and to discuss about symptoms, diagnosis and treatment.

**Case presentation:**

Both women few days after CS had surgical site infection, fever and elevation of lab inflammation markers. bacterial culture of the wound drainage was positive for Escherichia Coli and Enterococcus Faecalis.

**Conclusions:**

Fistula between uterine and skin is a rare condition but should be considered with signs of inflammation after cesarean section; 2D/3D ultrasound evaluation appears to be useful for diagnosis of UCFs when combined with CT fistulography and MRI in order to obtain early diagnosis and, consequently, a conservative surgery.

## Introduction

A fistula is a communication between two cavitated organs. Fistolization may occur after traumas, different injuries like overheating with wall necrosis but also after infections.

Although uterovesical, uterocolonic fistulae are not uncommon, uterocutaneous fistula (UCF) is actually a rare entity. By definition UCF consists in the communication between uterine cavity and skin and there are only a few reports of UCFs in the existing literature. Due to the rarity of this complication, the occurrence in an inflammatory or thrauma context with distortion of the normal anatomy, the need of preserving fertility, the treatment of UCFs is challenging even for experienced operators.

In this paper we describe two cases of UCFs after CS as result of infection of the surgical site by Escherichia Coli and Enterococcus Faecalis and report a review of literature.

## Case presentation

### Case report 1

A 36-year-old woman (gravida 1, para 1) referred to our department with vaginal abundant yellowish discharge after her first CS; a discharge with similar characteristics cames out from a hole in the cutaneuous suture. The existence of UCF was then suspected.

**Figure 1:**
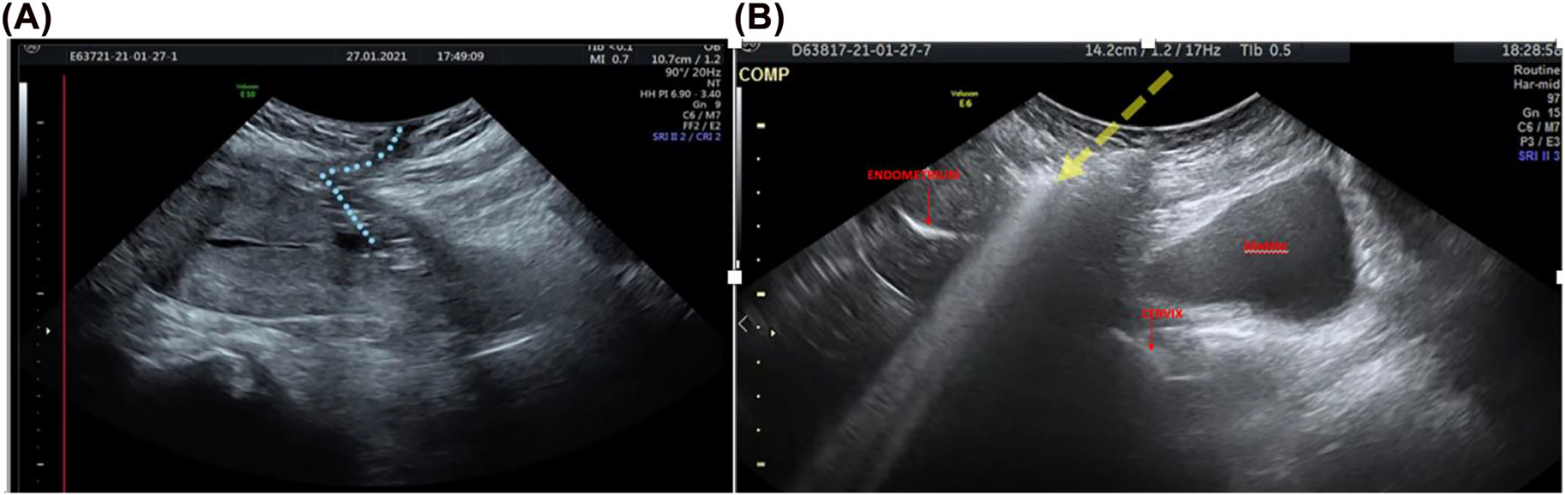
Case report 1. (A) Transabdominal US sagittal scan of uterus showing hypoecogenic fistula (dashed celestial line) between uterine cavity (isthmus) and anterior abdominal wall. (B). Transabdominal US sagittal scan of uterus after insufflation of hydrogen peroxide from the skin hole (yellow arrows) showing the passage in both the endometrial and cervical cavities.

She had cesarean section one month ago at a different hospital with no complications. The induction of labor in the 41st week of pregnancy failed so an urgent cesarean section was performed as the cardiotocographic trace worsened; amniotic fluid appeared poltaceous but the baby was healthy (Apgar 8–10, weight 3,200 g). On postoperative day 16 general conditions of the woman worsened and she was re-hospitalized for infected wound. An intravenous empirical antimicrobial therapy with Ceftriaxone 2 g daily was started. Her general conditions improved but suddenly an abundant genital discharge appeared; the woman concerned about her conditions decided to refer our department. General conditions were good, with no fever, blood panel showed normal white blood cells count and low C-reactive protein (CRP) level. The abdominal scar was regular with exception of a little hole on the right side from which purulent secretion came out. Gram stains, cultures and antimicrobial susceptibility testing of the discharge were performed. An iv empirical antimicrobial therapy with piperacillin-tazobactam 4.5 g three times a day and tigecycline 50 mg two times a day combined with clindamycin 600 mg was immediately started. In order to confirm the diagnostic suspect of UCF imaging investigations were performed. In detail, transabdominal and transvaginal ultrasound (GE Voluson E10, Kretz, US Machine) showed ventrofixation of the uterus and an irregularly shaped, not homogeneously hypoechogenic mass of 22 × 15 mm in size in correspondence of uterine isthmus at the level of previous CS scar, and distant 4, 2 mm from internal cervical os. This area was in continuity with a hypoechoic tubular area which ended into the abdominal wall (Figure 1A). In order to confirm the existence of a connection between abdominal wall and uterine cavity, 10 mL of hydrogen peroxide were slowly injected into the hole of the cutaneous scar and passage of fluid in the uterus was visible and both the endometrial and cervical cavity appeared hyperechoic (Figure 1B) as well as the duct-like structure localized between the skin and the uterine cavity. By placing a vaginal speculum, hydrogen peroxide appeared to come out from the external cervical os.

In order to define the size of the communication and the anatomical relationships a computed tomography (CT) fistulography, by injecting the contrast medium in the skin, was performed; this revealed fluid collection in a suprafascial cavity and passage of liquid troughout the abdominal wall until the anterior wall of the uterus and subsequent passage into the uterine cavity.

**Figure 2:**
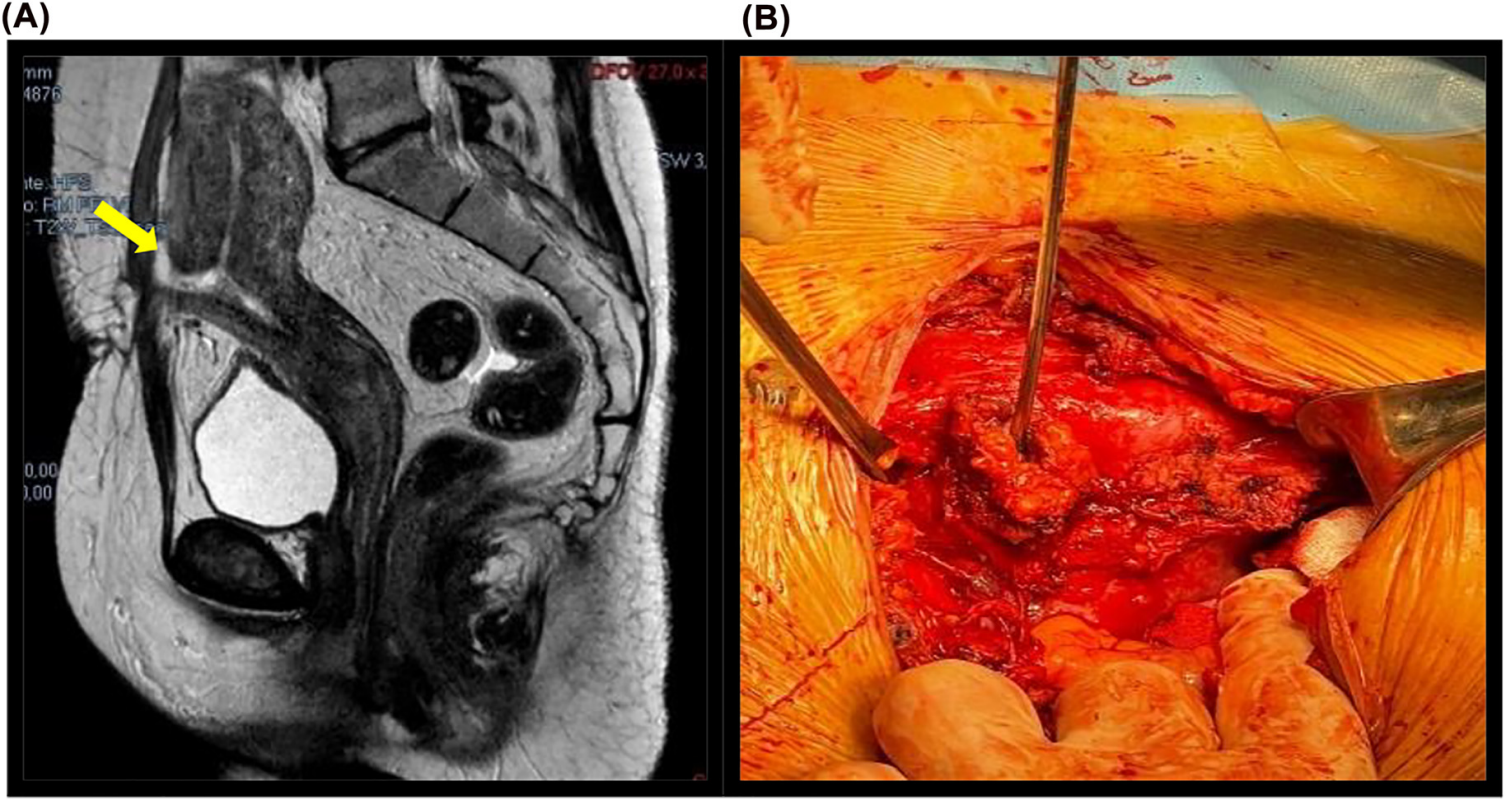
Case report 1. (A) Sagittal MRI scan of the patient showing the fistolous tract (yellow arrow) between uterine cavity and anterior abdominal wall. (B) Surgery: fistulous tract opening into the anterior wall of the uterine cavity.

Magnetic resonance imaging (MRI) also confirmed CT scan finding (Figure 2A).

The bacterial culture of the wound drainage was positive for *Escherichia coli* and *Enterococcus faecalis* which both were sensitive to the selected antibiotic therapy.

As the patients wanted to preserve the uterus she accepted to undergo reconstructive surgery after a month and a half from the cesarean section at the end of the diagnostic process. Surgery consisted in re-laparotomy, wound drainage, revision and debriedment, isolation of different layers, isolation of the fistula (Figure 2B) and removal of the fibrotic tissue, adhesiolysis of the anterior wall of the uterus, separation of the vescical wall and reconstruction of the hystmus. Intraabdominal and suprafascial drainages were left in place. Histopathology of the fistula showed the tract lined by granulation. The patient had an uneventful postoperatrive period and was discharged four days later. The women 2 months after the operation returned to be normally cycling.

### Case report 2

**Figure 3:**
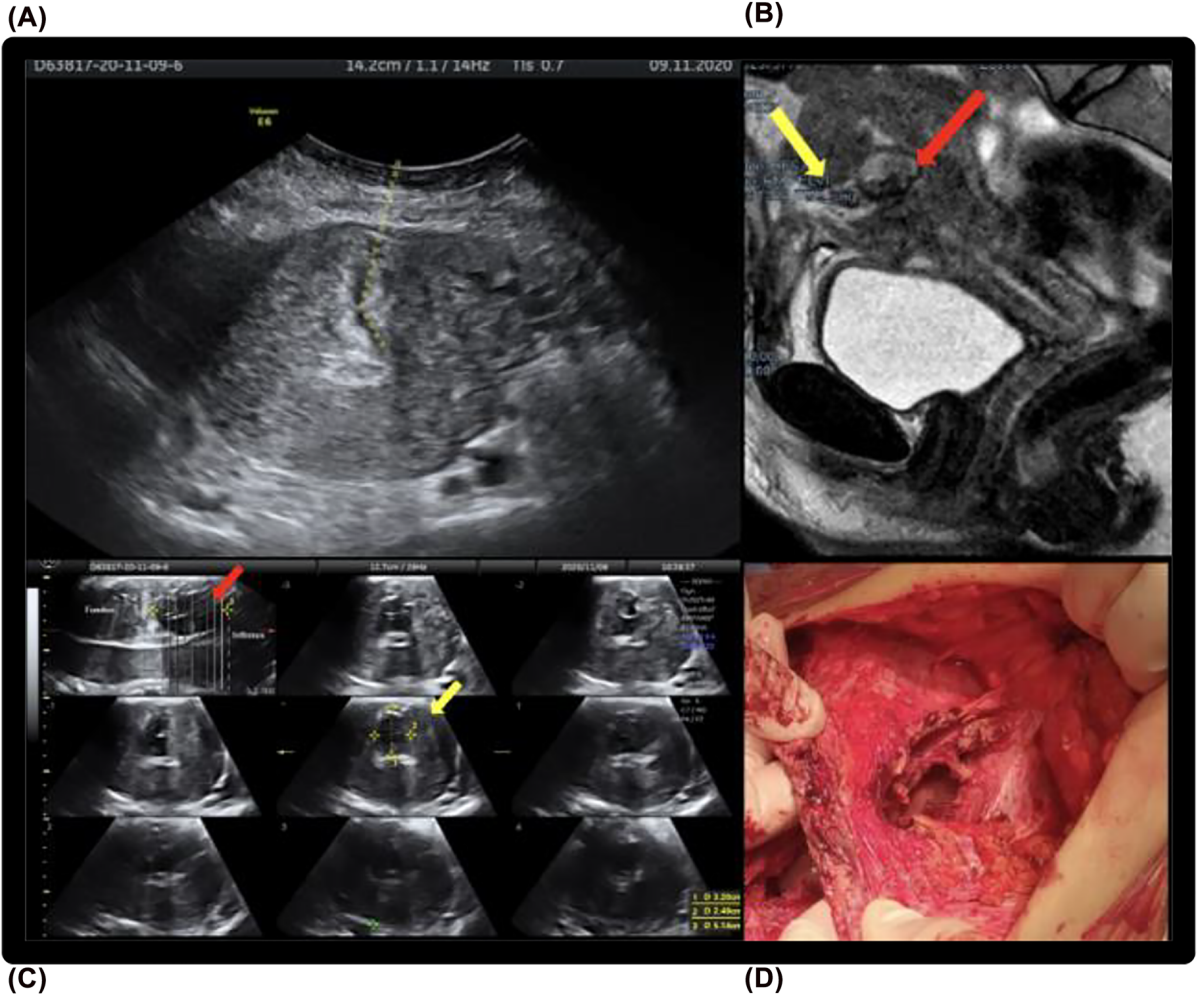
Case report 2. (A) Transverse ultrasound scan showing hypoechogenic fistulous tract between uterine cavity and anterior abdominal wall. (B) TUI 3D transabdominal US showing the major diameters of the fistulous tract: length equal to 5.14 cm in first sagittal plane image (red arrow); height of 3.20 cm and width of 2.49 cm in the axial plane of central image (yellow arrow). (C) Sagittal MRI scan of the patient showing the fistolous tract (yellow arrow) between uterine cavity and anterior abdominal wall; inferior to the fistula placental remnants are evident (red arrow). (D) Surgery: large fistula between anterior wall of uterus and the subcutaneous tissue of anterior abdominal wall at level of Pfannenstiel incision.

A 39-year-old woman (gravida 2, para 2) referred to our department with the diagnosis of an UCF following a CS. She suffered from essential hypertension. She had cesarean section one month ago at another hospital with no early complications and she was discharged in 3rd post-operstive day. On day 10 her general condition worsened and she was rehospitalizated for fever above 38 °C and secretion of pus through the CS scar. Computed Tomography revealed fluid collection with multiple bubbles of gas in the lower abdomen with a communication between uterine cavity and anterior abdominal wall through a 6 mm defect at the C-section incision site. The precence of placental remnants in the lower uterine segment was also detected without signs of adhesions and not associated with fistula. She referred to our clinic. Blood panel showed a white blood cells count within the normal range and a low level of the C-reactive protein (CRP). The patients underwent the same diagnostic procedures as above described but in this case hydrogen peroxide-enhanced transabdominal ultrasound reported progression of the fluid only in endometrial cavity and no through the cervical canal and vagina. Transabdominal ultrasound showed hypoechogenic fistulous tract between uterine cavity and anterior abdominal wall (Figure 3A). Moreover, transabdominal 3D TUI (Three-Dimensional Tomographic Ultrasound Imaging, using GE Voluson E10 Ultrasound Machine), reported the major diameters of the fistolous tract (5.14 cm in length in the sagittal plane, 3.20 cm in height and 2.49 cm in width in the axial plane) (Figure 3B) and the distance between the internal cervical os and fistolous tract, equal to 8.5 mm.

The bacterial culture of the wound drainage was also in this case positive for Escherichia Coli and Enterococcus Faecalis. The empirical antibiotic treatment was adjusted according to the wound drainage culture and susceptibility testing results, and ampicillin 2 g four times a day was added.

On MRI, a fistula tract was visualized between anterior wall of uterus and the subcutaneous tissue of anterior abdominal wall at level of Pfannenstiel incision; the presence of placental remnants in the lower uterine segment was also reported (Figure 3C).

The patient was then operated after a month and a half from the cesarean section at the end of the diagnostic process. Differently from case 1 she required hysterectomy, due to the concomitant finding of fibromatous uterus and salpingitis. Intraoperatively, after performing adhesiolsysis and debridement, the isthmic area was cleaned and inflammatory tissue removed successfully (Figure 3D) so that it was possible to perform reconstruction of the isthmus. However, basing on patient request total abdominal hysterectomy was performed. Pathological examination showed foreign body reaction, inflammatory necrosis of the uterine muscle, and a fistula tract in the muscular wall of the uterus with granulation tissue formation, hemorrhage, and fibrin deposition. Also, histopathological examination showed bilateral salpingitis and several uterine fibroids with various dimensions.

The postoperative course was uneventful and the patient was discharged on postoperative day 6.

## Discussion

Caesarean sections decrease the rates of peri-natal mortality and morbidity whenever indicated. However, CS is associated with increased incidence of postpartum maternal morbidity, particularly infection. Reported infections include endometritis, pelvic abscess, infected hematoma, peritonitis, pneumonia, urinary tract infection and superficial wound infection. Antibiotic prophylaxis has been found to reduce significantly the rate of wound infection following cesarean delivery and it is recommended for all CS deliveries.

However, notwithstanding antibiotic prophylaxsis, post-surgical infection may occur and result in severe complications like the occurrence of a UCF. This rare complication does not appear in the immediate postoperative days but requires some days in order to create a connection between the skin and the uterine cavity. Women are generally at home and report sudden hyperpyrexia, pain and increase or *de novo* appearance of vaginal discharge so that rehospitalization is required. Most frequent microorganisms detected in wound infections following CS include *Staphylococcus spp*, *Enterococcus faecalis*, *Escherichia coli*, *Proteus mirabilis* and *Pseudomonas species* [1].

In both cases described perioperative antibiotic prophylaxis was performed and clinical manifestation of the UCF occurred more than 2 weeks after surgery.

As regard as etiological agent interestingly in both cases the coltures were positive for *Escherichia coli* and *Enterococcus faecalis*. Enterococci are bacteria commonly living as saprophytes in the gastro intestinal tract, nevertheless sometimes they can be also isolated from polymicrobial cultures of soft tissue as well as intra-abdominal and pelvic cavity. Infection of wounds and abscesses caused by enterococci are commonly reported and associated with blood diffusion [2] resulting in serious infections and often life-threatening diseases including sepsis, endocarditis and meningitis.

UCFs may have different causes and usually result from post-partum or postoperative complications [3]. Placenta remnant [4] repeated previous abdominal operations, long-term stay of drains, technical reasons like incomplete closure of uterine incision, inflammation and wound dehiscence. Additional causes of UCF reported in literature include IUD migration or infection by actinomyces [5], uterovaginal malformation [6], false route at curettage, complicated vaginal delivery, or use of forceps. UCF formation due to endometriosis [7] and tuberculosis [8] were also described.

UCFs need adequate investigation. Transvaginal and TA ultrasound with saline injection, provided reliable evidence of the fistula. Test with hydrogen peroxide turned out to be a cheap and reliable test; 3D TUI defined the localization and the measures of fistula. More precise definition of fistulous route may be obtained by TC or RM fistulography [3].

In both our cases ultrasonography was useful to formulate the diagnosis of uterocutaneous fistula and to study its characteristics. Ultrasound offers many advantages: it is generally painless, widely accessible and less expensive than other methods, patients are not exposed to ionizing radiation making the procedure safer than other diagnostic techniques such as X-rays and CT scans and it does not require injection of intravenous contrast.

As regard as treatment previous reports were oriented to long-term medical treatment with GnRH analogue with the aim to delay surgery or to demolitive surgery and hysterectomy [2, 9, 10]. Our experience says that with an appropriate antibiotic treatment it is possible to limit the tissue damage so that reconstructive surgery may be a reliable option at the time of diagnosis. Accordingly, recent studies suggest that combined surgical and medical treatments may reduce the risk of hysterectomy [9].

In both cases the manifestation of UCF occurred late (15–30 post-operative day) after surgery when the patient was already discharged. This highlights the importance for clinicians to advise women with even mild infectious complications of the surgical wound to keep in touch with the structure after discharge and to control women strictly at least in the first month.

## Conclusions

UCF is a rare complication of CS but its occurrence should be taken in consideration in women with even mild infectious complications of abdominal wound. The occurrence of UCFs must be considered in women even after one month after CS when, in concomitance of worsening of general conditions, discharge from the cutaneous scar and vagina appears. Basing on our experience and on data from literature the demonstration of enterobacteria at cultures must be seen as a risk factor for severe complications. Proper investigation and imaging examination are needed for a correct treatment. Reconstruction and preservation of the uterus is very challenging but timely appropriate antibiotic therapy and accurate surgery permit to successfully reconstruct uterine integrity and functionality.
